# Supplementary material for: Electrospray-assisted cryo-EM sample preparation to mitigate interfacial effects
Source: Nat Methods. 2024 Apr 25;21(6):1023–32. doi: 10.1038/s41592-024-02247-0 (PMC11166575; doi:10.1038/s41592-024-02247-0)
Supplement: Supplementary file 1 — Supplementary Fig. 1 and Tables 1–3. [file 41592_2024_2247_MOESM1_ESM.pdf]

nature methods

Article

https://doi.org/10.1038/s41592-024-02247-0

Electrospray-assisted cryo-EM sample preparation to mitigate interfacial effects

In the format provided by the authors and unedited

Table of Contents Overview

Supplementary Figure and Tables

Supplementary Figure 1. Camera inspection of grid surface showing deposition of sprayed droplets.

Supplementary Table 1. ESI-cryoPrep cryo-sample preparation conditions

Supplementary Table 2. Comparisons of ESI conditions in MS analysis and cryo-EM sample preparation

Supplementary Table 3. Cryo-EM data-collection and processing statistics

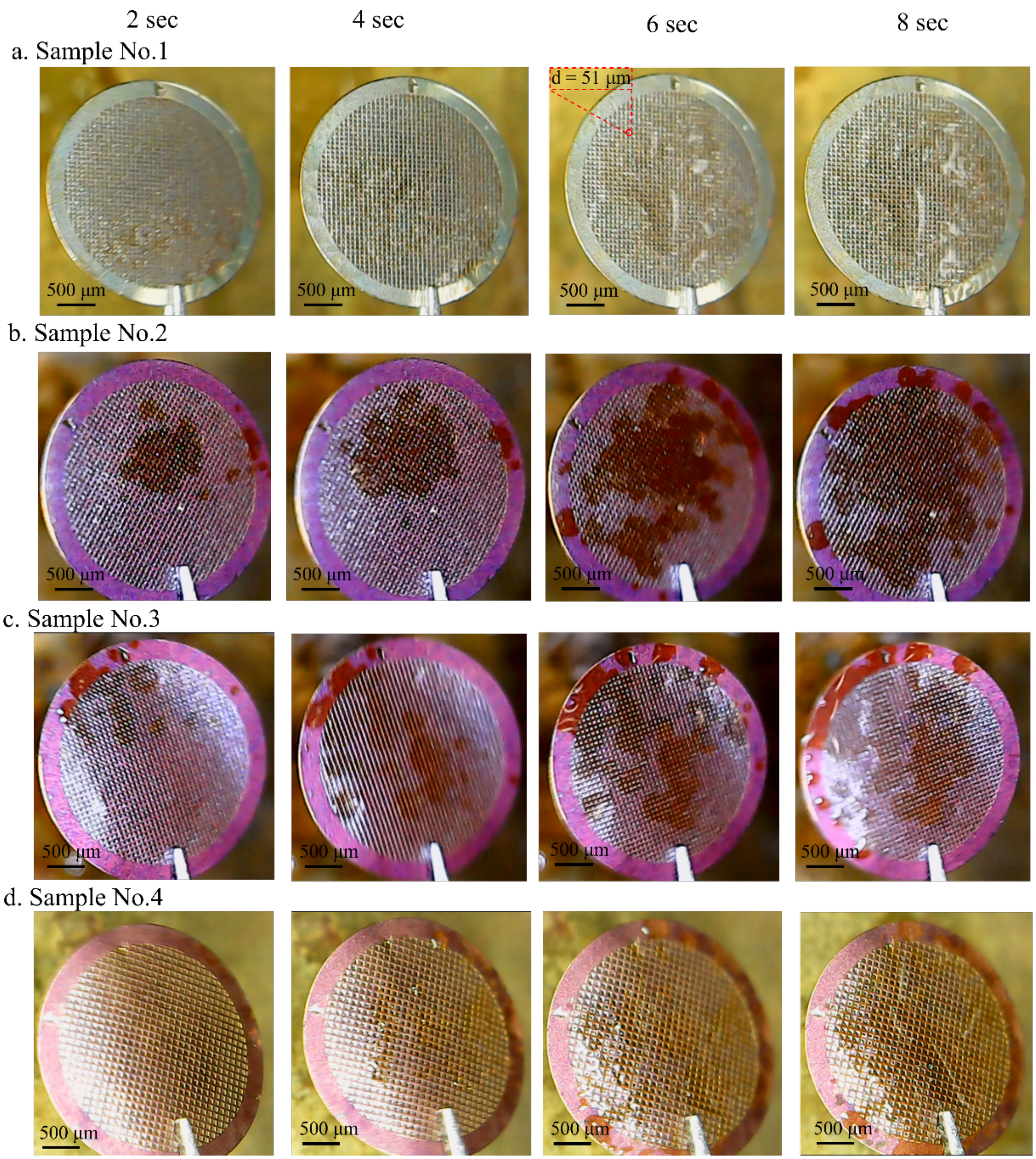

Supplementary Figure 1. Camera inspection of grid surface showing deposition of sprayed droplets. The spray processes were recorded at 2 sec, 4 sec, 6 sec, and 8 sec by a high frame-rate CCD camera. **a-d**. Droplets were collected in four different grids.

Supplementary Table 1. Cryo-sample preparation conditions

	70S ribosome	20S proteasome	apo-ferritin	ACE2	Streptavidin
Purified protein buffer conditions	20 mM HEPES-KOH, pH 7.6,<br>10 mM $Mg(OAc)_2$<br>30 mM KCl<br>7 mM beta-ercaptoethanol	50 mM Tris-HCl, pH 8.0,<br>100 mM NaCl	PBS buffer (50 mM NaH_2PO_4, pH 7.4, 150mM NaCl,)	HBS buffer (10 mM HEPES, pH 7.2, 150 mM NaCl)	140 mM NaCl,<br>8 mM Sodium Phosphate, 2 mM Potassium Phosphate, 10 mM KCl, pH 7.4
Dilution factor	30×	5×	10×	5×	8×
Dilution buffer	20mM Tris, pH 8.0, 100mM NH_4Ac				
Cryo-sample protein concentrations	0.44 µM	0.58 µM	1.2 µM	4 µM	2.5 µM
Molecular weight	2.5 MDa	750 kDa	444 kDa	70 kDa	52 kDa

Supplementary Table 2. Comparisons of ESI conditions in MS analysis and cryo-EM sample preparation

ESI components	MS analysis	Cryo-EM sample preparation
Capillary	4000-5000 V	3000-4000 V
Nebulizer	0.7 mBar	0 mBar
Desolvation dry gas	0-6 L/min	0 L/min
Desolvation temperature	20-50 °C	Room temperature
Sample flow rate	0.5-5 µl/min	0.3-0.5 µl/min

Supplementary Table 3. Cryo-EM data-collection and modeling statistics

	70S ribosome	20S proteasome	Apo-ferritin	ACE2	Streptavidin
EM data collection/processing					
Microscope	FEI Titan Krios	FEI Titan Krios	FEI Titan Krios	FEI Titan Krios	FEI Titan Krios
Voltage (kV)	300	300	300	300	300
Camera	Gatan K3	Gatan K3	Gatan K3	Gatan K3 with GIF Quantum (20 eV slit)	Gatan K3 with GIF Quantum (20 eV slit)
Mode	Counting	Counting	Counting	Counting	Counting
CS (mm)	2.7	2.7	2.7	2.7	2.7
Magnification	29 k×	29 k×	29 k×	105 k×	165 k×
Pixel size (Å)	0.97	0.97	0.97	0.8374	0.5191
Defocus series (μm)	-0.7 ~ -1.2	-0.7 ~ -1.2	-0.7 ~ -1.2	-1.0 ~ -1.5	-0.8 ~ -1.3
Exposure time (s)	2.56	2.56	2.56	1.28	0.43
No. of fractions	32	32	32	32	32
Exposure rate (e^- per pixel per second)	18.4	18.4	18.4	27.4	31.3
Total exposure ($e^-Å^{-2}$)	50	50	50	50	50
EM data processing					
Software	RELION-3.1, RELION-4.0, RELION-5.0, CryoSPARC-4.2				
No. of micrographs	1606	1801	1168	1793	827
No. of particles (contribute to final map)	77,872	283,047	158,045	137,545	218,178
Symmetry	*C1*	*D7*	*O*	*C1*	*D2*
Resolution (global) (Å)	2.77	2.04	2.15	3.28	1.95
Local resolution range	2.5-4.7	1.9-2.6	2.0-2.4	3.2-4.1	1.9-2.3
Sharpening B factor ($Å^2$)	50	62	68	100	52
