# Supplementary material for: Electrospray-assisted cryo-EM sample preparation to mitigate interfacial effects
Source: Nat Methods. 2024 Apr 25;21(6):1023–32. doi: 10.1038/s41592-024-02247-0 (PMC11166575; doi:10.1038/s41592-024-02247-0)
Supplement: Supplementary file 2 — Reporting Summary [file 41592_2024_2247_MOESM2_ESM.pdf]

nature portfolio

Corresponding author(s): Hong-Wei Wang

Last updated by author(s): Feb 18, 2024

Reporting Summary

Nature Portfolio wishes to improve the reproducibility of the work that we publish. This form provides structure for consistency and transparency in reporting. For further information on Nature Portfolio policies, see our Editorial Policies and the Editorial Policy Checklist.

Statistics

For all statistical analyses, confirm that the following items are present in the figure legend, table legend, main text, or Methods section.

n/a	Confirmed	
☐	☒	The exact sample size (*n*) for each experimental group/condition, given as a discrete number and unit of measurement
☐	☒	A statement on whether measurements were taken from distinct samples or whether the same sample was measured repeatedly
☒	☐	The statistical test(s) used AND whether they are one- or two-sided<br>*Only common tests should be described solely by name; describe more complex techniques in the Methods section.*
☒	☐	A description of all covariates tested
☐	☒	A description of any assumptions or corrections, such as tests of normality and adjustment for multiple comparisons
☐	☒	A full description of the statistical parameters including central tendency (e.g. means) or other basic estimates (e.g. regression coefficient) AND variation (e.g. standard deviation) or associated estimates of uncertainty (e.g. confidence intervals)
☒	☐	For null hypothesis testing, the test statistic (e.g. *F*, *t*, *r*) with confidence intervals, effect sizes, degrees of freedom and *P* value noted<br>*Give P values as exact values whenever suitable.*
☒	☐	For Bayesian analysis, information on the choice of priors and Markov chain Monte Carlo settings
☐	☒	For hierarchical and complex designs, identification of the appropriate level for tests and full reporting of outcomes
☒	☐	Estimates of effect sizes (e.g. Cohen's *d*, Pearson's *r*), indicating how they were calculated

Our web collection on statistics for biologists contains articles on many of the points above.

Software and code

Policy information about availability of computer code

Data collection	Cryo-specimens were prepared with ESI-cryoPrep method. Single-particle cryo-EM datasets were collected using AutoEMation2.0 software written by Dr. Jianlin Lei at Tsinghua University, and SerialEM (version 3.8 and 4.0). Dose symmetric tilt-series were collected with SerialEM (version 3.8 and 4.0) software.
Data analysis	Pre-processing of SPA and tomography dataset were performed to facilitate micrograph-selecting with the facility's homemade wrapper TsinghuaSoftwares.py and a homemade script Pre-process-tomo.sh incorporating MotionCor2 and Gctf, respectively. The beam-induced motion and radiation damage of cryo-EM micrographs were corrected with MotionCor2 (version 1.6.3). Particle picking was performed by template matching in RELION (version 3.1, 4.0 and 5.0) and CNN-based automatic picking in Topaz (version 0.2.4). The 2D and 3D analyses were performed in RELION (version 3.1, 4.0 and 5.0) and cryoSPARC (4.2 and 4.4). The CTF values of motion-corrected micrographs were determined by CTFFIND4 (version 4.1.14) and Gctf (version 1.18). The structural analysis was performed in UCSF Chimera (version 1.17) and UCSF Chimera X (version 1.6). Validations of the local resolution were assessed through a wrapper in RELION (version 3.1) and ResMap (version 1.1.4). The coordinates were real-space refined in PHENIX (version 1.19) and adjusted in Coot (version 0.9.8). For cryo-ET analysis, tilt-series were aligned using IMOD (version 4.11) and EMAN2 (version 2.29) to reconstruct the 3D tomograms. Particle-picking and 3D reconstruction were analyzed using EMAN2 (version 2.29) and Dynamo (version 1.1). All these softwares are open-source.

For manuscripts utilizing custom algorithms or software that are central to the research but not yet described in published literature, software must be made available to editors and reviewers. We strongly encourage code deposition in a community repository (e.g. GitHub). See the Nature Portfolio guidelines for submitting code & software for further information.

Data

Policy information about availability of data

All manuscripts must include a data availability statement. This statement should provide the following information, where applicable:

- Accession codes, unique identifiers, or web links for publicly available datasets
- A description of any restrictions on data availability
- For clinical datasets or third party data, please ensure that the statement adheres to our policy

The following publicly available data were used in the manuscript: apo-ferritin control dateset EMPIAR-10424. Crystal structures determined in previous work were aligned and fit to 3D maps in ChimeraX and then real-space refined in PHENIX for 70S ribosome (PDB IDs-6W6P), 20S proteasome (PDB IDs-1PMA), apo-ferritin (PDB IDs-1FHA), ACE2 (PDB IDs-1R42), and streptavidin (PDB IDs-7dy0). All necessary data for evaluating the conclusions are provided in the paper and/or Extended Data Materials. The coordinates of the ACE2 cryo-EM structure were deposited in the Protein Data Bank under accessions 8JWH. The corresponding cryo-EM map was deposited in the Electron Microscopy Data Bank (EMDB) under accessions EMD-36683.

Human research participants

Policy information about studies involving human research participants and Sex and Gender in Research.

Reporting on sex and gender	not applicable
Population characteristics	not applicable
Recruitment	not applicable
Ethics oversight	not applicable

Note that full information on the approval of the study protocol must also be provided in the manuscript.

Field-specific reporting

Please select the one below that is the best fit for your research. If you are not sure, read the appropriate sections before making your selection.

☒ Life sciences ☐ Behavioural & social sciences ☐ Ecological, evolutionary & environmental sciences

For a reference copy of the document with all sections, see nature.com/documents/nr-reporting-summary-flat.pdf

Life sciences study design

All studies must disclose on these points even when the disclosure is negative.

Sample size

For TEM analysis, each condition was characterized with the number of micrographs more than 5, and tens and hundreds of particles from each micrograph were auto-detected to calculate the percentage of intact proteins. Sample sizes were determined according to the occurrence frequency in each condition state, with confidence interval error bars added.
For cryo-EM single particle analysis, five different biological specimens were imaged and analyzed, which are 70S ribosome, 20S proteasome, apo-ferritin, ACE2, and streptavidin. The five sample proteins, 70S ribosome, 20S proteasome, apo-ferritin, ACE2, and streptavidin, were reconstructed at final resolutions of 2.77 Å, 2.04 Å, 2.15 Å, 3.28 Å and 1.95 Å, estimated by the Fourier Shell Correction (FSC)=0.143 cutoff criteria with a number of 77872, 283047, 158045, 137545, and 218178 particles contributed to the final reconstructions, from 1606, 1801, 1168, 1793, and 827 micrographs, respectively. The overall size and dataset associated with high-resolution reconstructions have been widely acknowledged for characterizing structural determination, particle orientation distribution and exploring particle defocus ranges.
For cryo-EM single particle control data, 2821 apo-ferrtin micrographs from EMPIAR-10424 on suspending ice , 3275 apo-ferrtin micrographs, 2209 ACE2 micrographs from conventional sample preparation method on supporting films were analyzed and compared with the ESI-cryoPrep dataset. The sample sizes for datasets were determined using the entire dataset contributing to the ultimate high-resolution reconstruction.
For cryo-ET analysis, four datasets of 12, 8, 6, and 18 tomograms were analyzed for 70S ribosome, 20S proteasome, apo-ferritin, and ACE2, respectively. The overall sample size is commonly employed for characterizing particle spatial distribution.

Data exclusions

For cryo-EM reconstruction, particles classified into bad classes with poorly defined features were excluded. To calculate the ice thickness of sample holes, we set a criteria to measure the z-height range of 85% particles to exclude outliers.

Replication

To demonstrate the reproducibility of the method and its benefits, we tested five sample proteins encompassing a large range of molecular sizes. All trials and attempts went successful.

Randomization

Representative tilt-series and negative staining micrographs were taken in a randomized way, we stochastically sampled the areas to record. During 3D refinement in 3D reconstruction softwares, particles were randomly divided into two subsets and independently reconstructed to determine the final resolution.

Blinding	Blinding experiments were not applicable to the current study since the treatments between the control data and the experiment data are the same.

Reporting for specific materials, systems and methods

We require information from authors about some types of materials, experimental systems and methods used in many studies. Here, indicate whether each material, system or method listed is relevant to your study. If you are not sure if a list item applies to your research, read the appropriate section before selecting a response.

Materials & experimental systems

n/a	Involved in the study
☒	☐ Antibodies
☒	☐ Eukaryotic cell lines
☒	☐ Palaeontology and archaeology
☒	☐ Animals and other organisms
☒	☐ Clinical data
☒	☐ Dual use research of concern

Methods

n/a	Involved in the study
☒	☐ ChIP-seq
☒	☐ Flow cytometry
☒	☐ MRI-based neuroimaging
